# Transcriptome analysis revealed the mechanism of skeletal muscle growth and development in different hybrid sheep

**DOI:** 10.5713/ab.24.0269

**Published:** 2024-10-25

**Authors:** Mengyu Lou, Sihuan Zhang, Wangxin Yang, Shuang Li, Hongguo Cao, Zijun Zhang, Yinghui Ling

**Affiliations:** 1 College of Animal Science and Technology, Anhui Agricultural University, Hefei 230036, China; 2 Anhui Provincial Key Laboratory of Local Livestock and Poultry Genetic Resources Protection and Biological Breeding, Hefei 230036, China

**Keywords:** Heterosis, Hub Genes, Production Performance, Sheep, Transcriptome

## Abstract

**Objective:**

This study aimed to analyze the molecular mechanism of heterosis in East Friesian sheep×Hu sheep (EH) hybrid sheep and Suffolk×EH (SHE) hybrid sheep (*Ovis aries*).

**Methods:**

In this research, the growth performance data of Hu sheep (H), EH and SHE from birth to 8 months of age were analyzed. Three 8-month-old sheep of each of the three strains (9 sheep in total) were chosen and their longissimus dorsi muscles were collected for transcriptome sequencing. We verified the expression of seven differentially expressed genes (DEGs) by real-time quantitative reverse transcription polymerase chain reaction (RT-qPCR).

**Results:**

The results showed: (1) body weight and chest circumference of EH were significantly greater than H (p<0.05), except at 4 months of age. Body weight and chest circumference of SHE was significantly higher than EH (p<0.05), except at 6 months of age. (2) 310 DEGs were screened in the EH and H, gene ontology and Kyoto encyclopedia of genes and genomes showed DEGs were mainly concentrate on the categories of actin cytoskeleton, calcium binding, cGMP-PKG and mitogen-activated protein kinase (MAPK) signaling pathway, which correlating the development of skeletal muscle and energy metabolism. 329 DEGs were screened in the SHE and EH. DEGs were mainly enriched in extracellular matrix-receptor interactions and cell adhesion molecules. (3) Protein–protein interaction screening yielded five (*MYL2*, *TNNI1*, *TNNI3*, *MYH11*, *TNNC1*) and three (*SOX10*, *COL2A1*, *MPZ*) pivotal DEGs regulating muscle development in EH and SHE. (4) RT-qPCR test results were consistent with transcriptome sequencing.

**Conclusion:**

This study provides candidate genes for improving sheep growth traits. It provides a theoretical basis for analyzing the mechanism of muscle development in crossbred sheep.

## INTRODUCTION

Sheep meat is widely loved by consumers for its high protein content, low fat content and tender meat [1,2]. In recent years, mutton has become one of the main meats consumed by Chinese consumers, but the growing need for high-quality mutton has not been met [3]. Therefore, improving the production traits and meat quality of sheep is an issue we need to address.

Compared with pure breeding sheep, hybrid sheep showed the advantages of faster growth rate, less fat and cholesterol content, leaner meat and fresh er tenderness in production [4]. A study compared the meat quality and slaughter traits of Tibetan sheep and Tibetan sheep×Suffolk, found that the meat production traits of Sa-Tibetan hybrid sheep was greater than Tibetan sheep [5]. Zhu et al [6] found the growth, reproduction and slaughter performance of Wuhu hybrid sheep with Wugu sheep as male parent and Hu sheep (H) as female parent were significantly better than those of pure H. Moreover, studies have shown that three breeds hybridized sheep can significantly improve meat quality and increase the levels of crude protein and amino acids in skeletal muscle, which has the potential to cultivate and produce high-quality mutton [7]. Song et al [8] used Romney rams to cross with Small Tail Han sheep (STH), and selected hybrid F1 ewes with good traits to cross with Poll Dorset rams. The results displayed the growth traits and slaughter performance of the ternary hybrid offspring were significantly greater than those of the binary hybrid sheep. Du et al [9] crossed the Dorper sheep (DP) rams with the Hantan binary hybrid ewes, and found that the ternary hybridization showed significant heterosis.

With the advantages of four-season estrus and heat and humidity resistance, the H has become the preferred mother of choice for the development of the meat sheep industry in the southern region of China and even the whole country [10]. East Friesian sheep (E) is well-known breed of dairy sheep, with outstanding performance in milk production, reproduction and meat production, and has been widely used in various countries as a sire to enhance the milk-producing performance of local breeds [11]. To improve the lactation performance and meat production performance of H, in the early stage of this experiment, H was used as the female parent, and E was introduced for crossbreeding. After analyzing the data related to reproductive performance and lactation performance of E×H (EH), we found that EH still retained the excellent performance of one fetus and multiple lambs. The lambing rate was 229.51%, and the weaning survival rate was 92.14%, which was 88.29% higher than that of H. The total milk production of EH within 5 to 45 days was significantly higher than that of H (p<0.01), the lactation performance was significantly improved [12]. Studies have shown that compared with binary crosses, ternary crosses can give fuller play to the advantages of hybrid ewes and effectively utilize the hybrid advantages of individual ternary hybrids, so that the advantages of the three parental breeds are reflected in the offspring of ternary crosses. Therefore, based on the EH, this experiment introduced the world-famous mutton sheep variety Suffolk ram to crossbreed with EH, which was expected to further enhance the meat productivity and reproductive performance of the hybrid sheep [13].

However, the exploration of heterosis and the mechanism of heterosis in EH binary hybrids and Suffolk sheep (S)×EH (SHE) ternary hybrids has not been reported. In this paper, we analyzed the data related to the production performance of five stages of H, H×E and SHE from birth to 8 months of age. The bioinformatics analysis of the longissimus dorsi muscle was carried out by RNA-Seq, to provide reference for exploring the key genes and functional pathways regulating the meat productivity of binary hybrid sheep and ternary hybrid sheep.

## MATERIALS AND METHODS

### Animal feeding and sample collection

**Figure 1:**
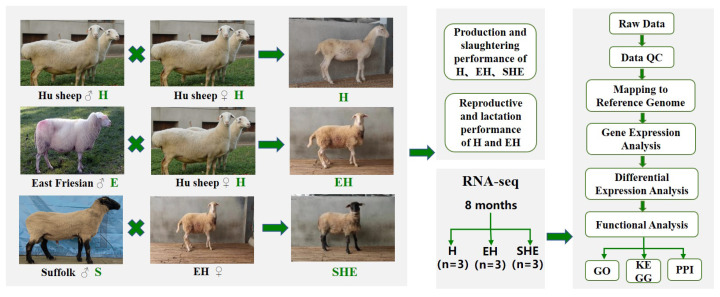
Schematic diagram of hybridization method. GO, gene ontology; KEGG, Kyoto encyclopedia of genes and genomes; PPI, protein–protein interaction.

In this research, purebred H, E×H and S×EH were used as the research objects (Figure 1). All sheep were raised under similar management conditions. The body weight, body height, body length, chest circumference and cannon circumference of sheep at 2 days, 2, 4, 6 and 8 months of age were measured under fasting condition.

Animal protocols were approved by the Review Committee for the Use of Animal Subjects of Anhui Agricultural University (No. AHAU20101025). Three 8-month-old sheep of each of the three strains (9 sheep in total) were randomly selected, fasted for 12 h and watered for 3 h before slaughter, euthanized after intravenous injection of barbituric acid (30 mg/kg), carcass weight, meat weight, carcass fat content (GR) value, and eye muscle area were measured. The longissimus dorsi muscle was collected as a test sample, rinsed three times with phosphate-buffered saline (Dulbecco’s phosphate-buffered saline) containing 1× penicillin and streptomycin, and then the samples were immediately placed in a liquid nitrogen tank (East Asia Industry and Trade Co., Ltd., Leshan, China). Frozen and transferred to a refrigerator at −80°C until RNA extraction.

### Total RNA extraction, library construction and sequencing

The longissimus dorsi muscle samples were ground into powder and added to Trizol (Adderall Biologicals, Beijing, China) to extract total RNA. RNA contamination and degradation were examined by 1.5% agarose gel electrophoresis. The purity and concentration of RNA were determined by Nanodrop One Micro UV-visible spectrophotometer (Thermo Fisher Scientific, Waltham, MA, USA). Finally, integrity and total amount of RNA were accurately determined by Agilent2100 biometric analyzer.

The 5 μg of RNA from each sample was used to build the library. mRNA with polyA tail was enriched using Oligo (dT) magnetic beads. Double-stranded cDNAs were synthesized from dNTPs, and the purified cDNAs were subjected to end repair and addition of polyA junctions, respectively. The cDNAs around 250 to 300 bp were screened for polymerase chain reaction (PCR) amplification to construct cDNA libraries. The library construction and sequencing (NovaSeq 6000; Illumina, San Diego, CA , USA) were done by Beijing Novogene.

### Quality control and transcript assembly

The raw data were filtered using fastp (version 0.19.7) software as follows: adaptor reads; unknown base information reads; and reads for which the number of bases with Qphred ≤20 accounted for more than 50% of the entire read length. Finally, we calculated and analyzed the Q20, Q30, and guanine-cytosine content of the cleaning data.

The reference genome and gene annotation file (GCF_ 016772045.1) for sheep (*Ovis aries*) was downloaded from National Center for Biotechnology Information (NCBI). Indexes of reference genomes were constructed using HISAT2 (v2.0.5) and compared. The transcripts were then assembled using StringTie (1.3.3b).

### Screening of differentially expressed genes and functional annotation

Expression levels of genes are expressed as transcript fragments per kilobase per million mapped read values (FPKM). DESeq2 (1.20.0) was used to analyzed for differences. The differentially expressed genes (DEGs) screening criteria were as follows: p-value <0.05 and |log_2_ Fold Change|≥1.

The clusterProfiler (3.8.1) software was used to perform gene ontology (GO) functional enrichment analysis and Kyoto encyclopedia of genes and genomes (KEGG) pathway enrichment analysis on the DEGs. p-value <0.05 was set as significant enrichment, p-value <0.01 indicates extremely significant enrichment.

### Protein–protein interaction interaction analysis

The interactions between differential genes were identified in the STRING (11.5) database by blastx comparison, and the resulting interactions were imported into Cytoscape software for visualization of the interactions.

### Real-time reverse transcription quantitative polymerase chain reaction was used to verify the accuracy of the RNA-Seq

Total RNA was extracted from tissues using Trizol (Beijing Adelaide Biotechnology Co., Ltd., Beijing, China), and cDNA was synthesized using reverse transcriptase and oligo-dT primers according to the instructions of the manufacturer MonScript RTIII All-in-One Mix with dsDNase (Monad Biotech Co., Ltd., Suzhou, China). Primer pairs were designed using NCBI Primer-BLAST and synthesized by TsingKe Biotechnology (TsingKe, Nanjing, China). The *GAPDH* internal reference gene was used as a control (Table 1). Real-time reverse transcription quantitative PCR (RT-qPCR) was performed using CFX Connect RT PCR Detection System (Bio-Rad, Hercules, CA, USA). Reaction system (20 μL): 10 μL of 2×Q3 SYBR qPCR Premix (TOLOBIO, Shanghai, China), 8.2 μL of sterilized water, 1 μL of DNA template (cDNA solution) and 0.4 μL of RT-qPCR forward primer (10 μmol/L). Amplification conditions were as follows: pre denaturation at 95°C for 30 s followed by 40 cycles of 95°C for 10 s and 60°C for 30 s. Melting curve analysis was conducted at 95°C for 15 s, 60°C for 34 s, and 95°C for 15 s.

### Statistical analysis

The mRNA expression levels were calculated by 2^−ΔΔCt^. All data were expressed as the means±standard deviation and were analyzed by IBM SPSS Statistics v23.0. Statistical significance was assessed using T-test and one-way analysis of variance (ANOVA). Figures were generated by GraphPad Prism 8.0. p<0.05 indicates significant difference.

## RESULTS

### Comparison of growth and slaughtering performance of H, EH and SHE

**Figure 2:**
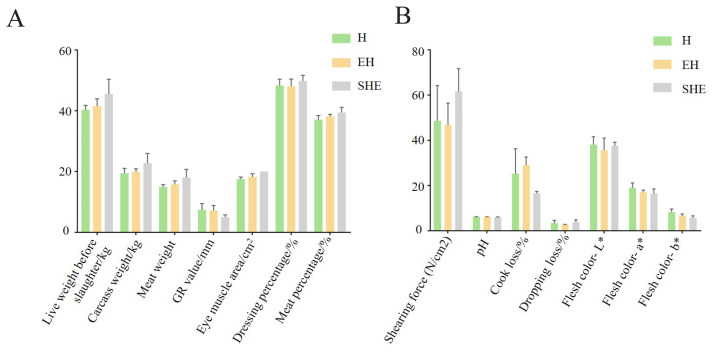
Slaughtering performance and meat quality of H, EH and SHE at 8 months of age. (A) Slaughter performance of H, EH and SHE. (B) Meat quality of H, EH and SHE. The green color indicates Hu sheep (H); the yellow color indicates East Friesian×Hu sheep (EH); the gray color indicates Suffolk sheep (S)×EH (SHE).

The production performance and slaughter performance of H, EH and SHE at 2 days, 2, 4, 6 and 8 months of age were measured. The results indicate in terms of growth performance, body weight and chest circumference were significantly (p<0.05) greater in EH than in H, except at 4 months of age. Body weight and chest circumference were significantly higher in the SHE than in the EH, except at 6 months of age (Table 2). The results showed EH had higher pre-slaughter live weight, carcass weight, net meat weight, eye muscle area and slaughter rate than H, and lower GR than H; SHE had higher pre-slaughter live weight, carcass weight, net meat weight, eye muscle area and slaughter rate and lower GR than EH. However, there was no significant difference in any of the slaughter performance indexes, it may be caused by too few samples (Figure 2). The study found the shear force and drip loss of EH were the lowest among the three sheep populations, indicating that the meat quality of EH was greater than H and SHE. In summary, the EH was superior to the H in terms of production performance, while the ternary crossbred SHE population was superior to the EH in terms of growth performance.

### Quality analysis of RNA-Seq data

After transcriptome sequencing, an average of 43.7 million, 46.06 million and 45.29 million raw reads were obtained for H, EH and SHE, respectively, which were screened to obtain 41.71 million, 43.29 million and 43.39 million reads, respectively. Among the three groups of sheep, the sequencing comparison rates were 96.36%, 96.42% and 96.30%, respectively. The overall sequencing error rates of the data were all below 0.03%, with Q20>96.54% and Q30>90.61%, indicating that the sequencing data were valid (Table 3).

### Screening for differentially expressed genes

**Figure 3:**
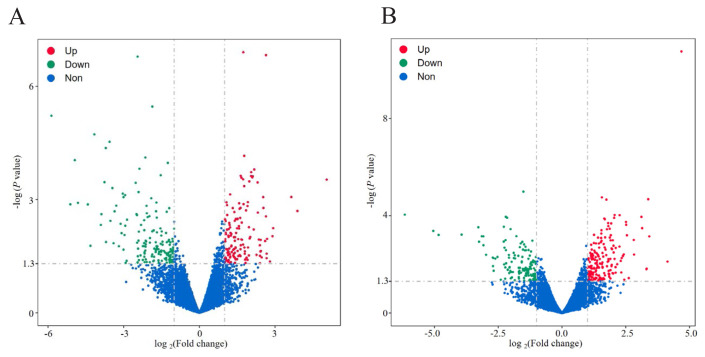
Volcano map of DEGs. (A) DEGs in H and EH. (B) DEGs in EH and SHE. The DEGs screening criteria were as follows: p-value <0.05 and |log2 Fold Change|≥1.The red indicates up-regulated DEGs; the green indicates down-regulated DEGs; the bule indicates no difference. DEGs, differentially expressed genes; H, Hu sheep; EH, East Friesian×H ; SHE, Suffolk sheep×EH.

310 DEGs (145 up- and 165 down-regulated) were identified in the EH and the H (Figure 3A), and the differential genes included *MYL2*, *TNNC1*, *TNNI1*, *TNNI3*, *MYH11*, etc. Muscle contraction is mediated by troponin, myosin, actin, calcium and adenosine triphosphate. *MYL2* and *TNNC1* are essential components of myosin and troponin, respectively [14]. Troponin I (TnI) is encoded by three homologous genes (*TNNI1*, *TNNI2* and *TNNI3*). Indispensable in the regulation of contraction and relaxation by calcium ions. *MYH11* participated in myoblast cell development, myotube differentiation and other regulatory processes in skeletal muscle development [15]. 329 DEGs (191 up- and 138 down-regulated) were identified in the SHE and the EH (Figure 3B), involving *SOX10*, *COL2A1*, *MPZ*, and *MYL3*, which are related to skeletal muscle development.

### Enrichment analysis of gene ontology and Kyoto encyclopedia of genes and genomes

**Figure 4:**
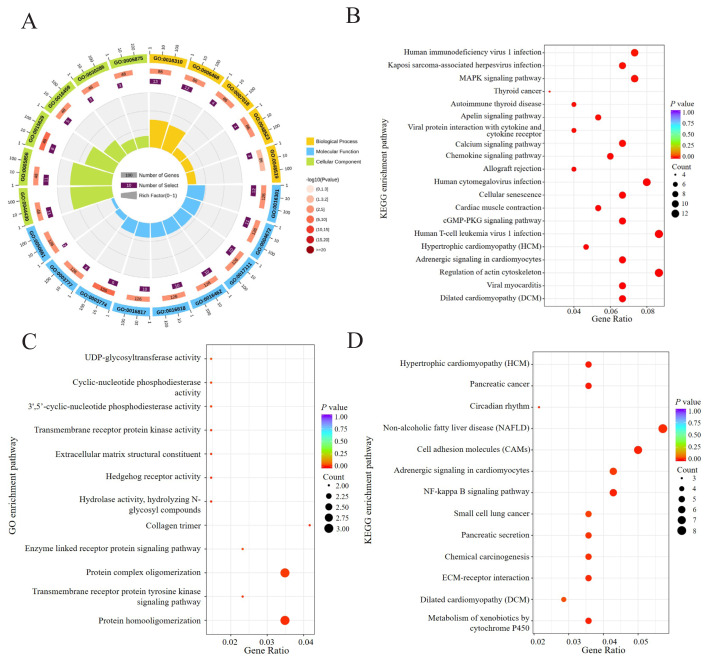
Functional enrichment analysis of DEGs. (A) Top 20 GO enrichment pathway of H and EH. (B) Top 20 KEGG pathways of H and EH. (C) All GO enrichment pathways of EH and SHE. (D) All KEGG pathways of EH and SHE. KEGG, Kyoto encyclopedia of genes and genomes; GO, gene ontology; DEGs, differentially expressed genes; H, Hu sheep; EH, East Friesian×H ; SHE, Suffolk sheep×EH.

To better understand the functions of these differential genes, GO and KEGG enrichment analyses of DEGs were performed in this experiment. GO annotation showed that the DEGs of EH and H were significantly enriched in 42 functional pathways, and many differential genes (such as *TNNI3*, *TNNI1*, *MYL2*, *MYH7B*, *TNNC1*) were enriched in actin cytoskeleton (p-value = 1.56E-07), organelle part (p-value = 0.012) and calcium ion binding (p-value = 0.031) related to skeletal muscle development (Figure 4A). KEGG enrichment analysis showed that the DEGs were significantly involved in 58 pathways, such as cGMP-PKG signaling pathway (p-value = 3.51E-04), MAPK signaling pathway (p-value = 2.88E-03), Wnt signaling pathway (p-value = 5.43E-03), cyclic adenosine monophosphate (cAMP) signaling pathway (p-value = 6.74E-03) and other pathways related to skeletal muscle energy metabolism (Figure 4B).

Enrichment analysis of DEGs between SHE and EH was carried out. GO annotation showed that 12 pathways were enriched in cellular components, molecular functions, biological processes, etc. Among them, collagen trimer (p-value = 0.012) and extracellular matrix (ECM) structural components (p-value = 0.017) were related to muscle development (Figure 4C). KEGG enrichment analysis showed the DEGs was significantly involved in 13 pathways, including cell adhesion molecules (p-value = 7.96E-03), ECM-receptor interactions (p-value = 0.012) and other pathways (Figure 4D), Cao et al [16] found that these two pathways were the two most enriched pathways in the development stages of Pekin and Hanzhong ducks and were closely related to muscle development.

### Protein–protein interaction interaction analysis

**Figure 5:**
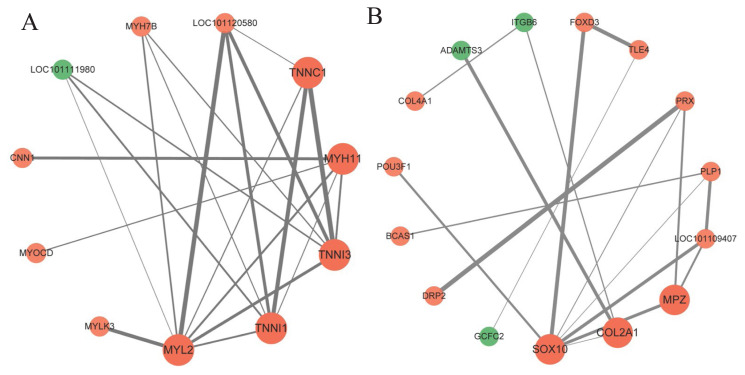
PPI interaction network diagram. (A) PPI interaction analysis of H and EH. (B) PPI interaction analysis of EH and SHE. Nodes represent proteins. Edges represent protein-protein associations. The relationship between the two proteins is expressed through the thickness of the line; the thicker the line, the closer the relationship. PPI, protein–protein interaction; H, Hu sheep; EH, East Friesian×H; SHE, Suffolk sheep×EH.

To further narrow down the candidate genes, protein–protein interaction (PPI) interaction analysis was performed on the DEGs. 68 genes were involved in PPI interaction in EH and H. Combined with functional enrichment analysis and literature reports, five hub genes closely related to skeletal muscle development were screened, including *MYL2* (node = 8), *TNNI1* (node = 6), *TNNI3* (node = 6), *MYH11* (node = 5) and *TNNC1* (node = 4) (Figure 5A). A total of 35 genes were involved in the PPI interaction between ternary hybrid sheep and binary hybrid sheep. Among them, *SOX10* (n = 7) was in the center of the PPI interaction network (Figure 5B). Studies have shown that *SOX10* has a role in regulating muscle development [17].

### Validation of transcriptome sequencing results by real-time quantitative reverse transcription polymerase chain reaction

**Figure 6:**
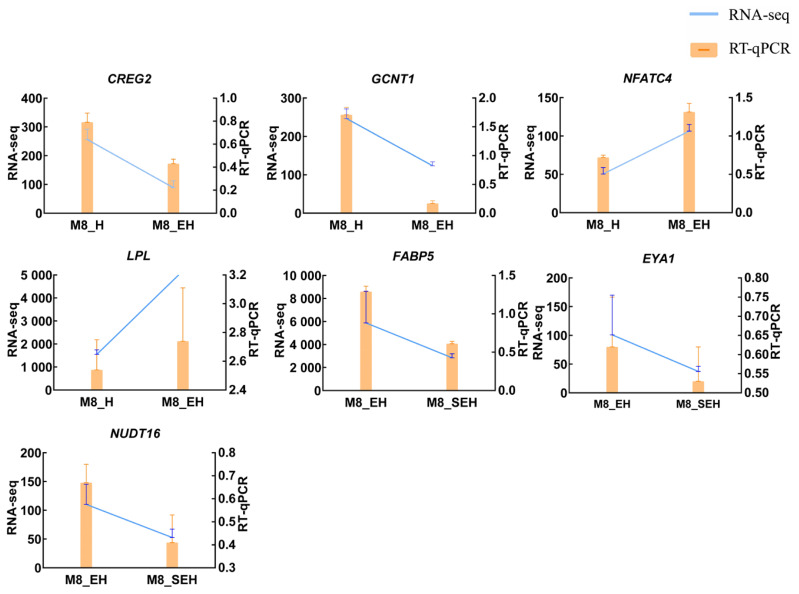
Comparison of RT-qPCR and RNA-Seq results of differentially expressed genes. The blue, line graph indicates RNA-Seq; the orange, bar graph indicates RT-qPCR. RT-qPCR, real-time quantitative reverse transcription polymerase chain reaction; M8_H, 8-month-old Hu sheep; M8_EH, 8-month-old East Friesian×H; M8_SHE, 8-month-old Suffolk sheep×EH.

Seven DEGs were randomly selected to verify the accuracy of RNA-Seq data by RT-qPCR (Figure 6). Expression trends of DEGs were equal with RNA-Seq results, indicating RNA-Seq data are reliable.

## DISCUSSION

Crossbreeding has become an effective tool to improve the growth rate, meat quality and production performance of sheep breeds [18]. Analyzing the mechanism of heterosis from a genetic perspective is essential for optimizing the production performance and meat quality traits of sheep [19]. In recent years, RNA-Seq has been applied to explore the mechanism of heterosis in hybrid sheep. Transcriptome analysis of the longissimus dorsi muscle of DP×STH and Mongolia (MG)×STH showed the meat quality of DP×STH sheep was better than that of MG×STH. And 13 key candidate genes affecting meat quality traits in sheep were identified [20]. Song et al [21] used RNA-Seq to screen out four candidate genes (*MSTR*, *IFRD1*, *PPARD*, *MYL2*) related to growth and meat quality of STH and the F1 generation of STH×S. In this study, we used transcriptome sequencing technology to bioinformatically analyze the longissimus dorsi muscle of 8-month-old H, EH and SHE, and to excavate the key genes and functional pathways regulating the improvement of production performance of hybrid sheep.

310 DEGs were screened in EH and H. GO and KEGG pathway enrichment analysis showed that most of the DEGs were significantly enriched in pathways closely related to muscle development and energy metabolism, such as actin cytoskeleton, calcium binding, MAPK signaling pathway and cAMP signaling pathway. PPI analysis showed that *MYL2, TNNI1, TNNI3, MYH11* and *TNNC1* genes were located at the core of the interaction network and were closely related to other genes.

Muscle fiber is an important part of skeletal muscle, which determines the ability of skeletal muscle contraction and metabolism, and is a key factor affecting muscle development and meat quality [22]. According to the method of energy metabolism, muscle fibers can be divided into oxidized and enzymatic, oxidized muscle fibers showed higher endurance and oxidative metabolic enzyme activity, belonging to slow muscle fibers, enzymatic muscle fibers contract quickly but have a short duration and are fast muscle fibers [23]. The quality of mutton will be seriously affected when many enzymatic muscle fibers increase in muscle [24]. *MYL2* is a myosin regulatory light chain that acts as a molecular motor to provide energy for muscle contraction. It has the function of regulating muscle fiber activity and movement, and is a typical slow muscle fiber molecule [25]. *TNNI1* is present in the sarcomere filaments of the striated muscle, playing an important regulator of striated muscle contraction. It regulates muscle contraction under the change of calcium concentration [26]. Previous studies have shown overexpression of *MYL2* and *TNNI1* genes promotes the expression of genes associated with muscle development [27,28]. *TNNC1* is the core regulatory protein of striated muscle contraction and the core component of slow muscle fibers, which has a positive effect on skeletal muscle myogenesis [25,29]. *TNNC1* and *TNNI1* are involved in actin-binding and glycolysis/glycogenesis pathways and play a central regulatory role in muscle fiber types [30]. *MYH11* is a myosin, affecting muscle fiber composition and muscle fat content [15]. The cytoskeleton is a cellular scaffold responsible for determining the shape and length of myofibers and transmitting forces generated by actin-myosin interactions to the extracellular fiber skeleton [31]. Ca^2+^ mediates excitation-contraction coupling in skeletal muscle, stimulates energy production in skeletal muscle mitochondria, and plays a crucial role in regulating slow muscle fiber formation in skeletal muscle [32]. Our bioinformatics analysis showed that *MYL2*, *TNNI1*, and *MYH11* were enriched in the actin cytoskeletal signaling pathway, and *TNNC1* was enriched in the calcium signaling pathway, all of them were up-regulated in EH, indicating that these genes are the key genes regulating the muscle development process of EH.

329 DEGs were screened in the SHE and EH. These DEGs were enriched into 12 GO functional pathways and 13 KEGG signaling pathways, including ECM-receptor interactions and cell adhesion molecules closely related to muscle development. PPI analysis showed that the *SOX10* gene is located at the core of the PPI network and closely interacts with *COL2A1* and *MPZ* genes.

*SOX10* belongs to the SOX family of transcription factors involved in embryogenesis, cell differentiation, and bone formation. It is an essential transcription factor for the development of neural crest cells (NCC) and peripheral nervous system (PNS) [33]. During the development of the body, NCC and skeletal muscle lines are established simultaneously, which provides a basis for the establishment of functional interaction between skeletal muscle cells and PNS in the later stage [34]. PNS and motor neurons play a role in regulating muscle development during late developmental neuromuscular junctions and muscle spindle formation [35]. Studies have shown *SOX10* deficiency resulted in a significant reduction in the number of Pax7+ muscle progenitor cells and MyoD+ differentiated myoblasts, severely interfering with myogenesis [18]. Muscle development is regulated by ECM-receptor interactions and cell adhesion molecule [20]. Myogenesis involves a series of complex processes such as proliferation of muscle progenitor cells, differentiation of myoblasts and formation of multinucleated myotubes [36]. Among them, cell adhesion molecules are involved in the normal processes of cell proliferation, differentiation, and apoptosis as cell-surface receptors, and are indispensable in the interaction of cells, tissues and extracellular matrix [37]. *MPZ* is a structural protein in the myelin sheath of the PNS that contributes to the transmission of nerve impulses and has also been shown to be involved in myocyte contraction, metabolism and cell repair [38]. In this experiment, *MPZ* was enriched in the cell adhesion molecule signaling pathway and involved in cell adhesion in this assay, consistent with previous findings. Reduced *COL2A1* protein expression may inhibit ECM-receptor interactions and ECM degradation [39]. Our bioinformatics analysis showed *COL2A1* was enriched in the ECM-receptor interaction pathway and was up-regulated in SHE, suggesting that *COL2A1* may regulate muscle development through ECM-receptor interaction pathway.

## CONCLUSION

In this experiment, we integrative analysis PPI networks and enrichment pathways, four (*MYL2, TNNI1, MYH11, TNNC1*) and three (*SOX10, COL2A1, MPZ*) pivotal DEGs regulating the growth performance of EH and SHE, respectively. The heterosis of crossbred sheep over purebred sheep for muscle and growth performance enhancement may be driven by these genes. These results contribute to a deeper understanding of the mechanisms of skeletal muscle formation, provide candidate genes for improved growth traits, and help improve growth and developmental traits in sheep through molecular breeding.

## Figures and Tables

**Table 1:** Primer information

Primer	Primer sequences (5′~3′)	Annealing (°C)	Product length (bp)
*GAPDH*	F: CCACGCCATCACTGCCACCC	62.5	249
	R: CAGCCTTGGCAGCGCCAGTA		
*CREG2*	F: AAGCCATGTTTTCAAGACACCCA	57.5	120
	R: TGTCGGCTACTCCCCCATAC		
*GCNT1*	F: CTGCCCAGTTGGAGACTCAT	58	101
	R: CATTCTGTGCCCATGAAGAGTG		
*NFATC4*	F: CCAATCAGAGACAGACCGGGAC	59	168
	R: GGTGGGGCGTCTTCTGAGTC		
*LPL*	F: CTTCAACCACAGCAGCAAAA	55	211
	R: AAACTTGGCCACATCCTGTC		
*FABP5*	F: CTCACCTGTCACGCTTGTCC	61	126
	R: AGCCATCCCCACTCCTACTT		
*EYA1*	F: TGGAATCTCCTCCTATGGCATC	57	135
	R: ATCTGGTAGCTGTACGGTGC		
*NUDT16*	F: ATGCGTAGGCTTGAGCTGG	60	145
	R: CAAAGCGCATCTGCATCAGC		

bp, base pair; *GAPDH*, glyceraldehyde-3-phosphate dehydrogenase; *CREG2*, cellular repressor of E1A stimulated genes 2; *GCNT1*, glucosaminyl (N-acetyl) transferase 1; *NFATC4*, nuclear factor of activated T cells 4; *LPL*, lipoprotein lipase ; *FABP5*, fatty acid binding protein 5; *EYA1*, EYA transcriptional coactivator and phosphatase 1; *NUDT16*, nudix hydrolase 16.

**Table 2:** Differences in production performance among H, EH and SHE sheep populations

Age	Variety	Body weight (kg)	Body height (cm)	Body length (cm)	Chest circumference (cm)	Cannon circumference (cm)
2 days	H	3.13±0.43^c^	37.58±1.84	27.39±1.49^b^	34.49±1.74^c^	5.41±0.42^b^
	EH	3.37±0.73^b^	37.29±2.58	27.3±2.46^b^	35.25±2.6^b^	5.56±0.53^b^
	SHE	4.06±0.84^a^	38.08±2.6	28.2±2.12^a^	37.3±2.54^a^	6.02±0.52^a^
2 months	H	16.18±2.14^c^	53.46±2.54^b^	54.64±2.47^c^	57.64±2.58^c^	7.87±7.96^a^
	EH	18.31±3.4^b^	55.32±3.21^a^	56.05±3.69^b^	59.23±4.26^b^	6.98±0.47^b^
	SHE	21.54±3.5^a^	56.02±2.96^a^	57.6±2.89^a^	63.65±3.6^a^	7.45±0.49^a^
4 months	H	25.02±4.23^b^	59.41±2.72	61.71±3.79^ab^	65.14±4.38^b^	6.76±0.5^b^
	EH	24.25±4.29^b^	58.36±3.72	60.22±3.06^b^	66.73±4.62^b^	6.74±0.52^b^
	SHE	30.22±3.84^a^	58.29±2.89	62.52±3.18^a^	71.21±4.91^a^	7.4±0.59^a^
6 months	H	29.27±3.78^b^	63.09±2.05^b^	66.11±2.91^b^	70.88±3.49^b^	7.17±0.38^b^
	EH	34.85±5.07^a^	65.59±2.42^a^	68.73±2.74^a^	79.78±4.13^a^	7.42±0.54^ab^
	SHE	37.55±3.41^a^	65.44±1.98^a^	68.57±2.26^a^	80.22±2.56^a^	7.69±0.39^a^
8 months	H	40.21±2.49^c^	67.07±2.21	70.93±2.47	80.41±3^c^	7.96±0.4^b^
	EH	43.32±3.99^b^	68.1±1.87	71.59±2.05	83.79±3.91^b^	8.4±0.46^a^
	SHE	46.27±3.73^a^	67.78±1.38	72.24±2.51	85.9±2.41^a^	8.62±0.38^a^

H, Hu sheep; EH, East Friesian×H; SHE, Suffolk sheep (S)×EH.

a–c Shoulder labels within the same age with different letters indicate significant differences (p<0.05).

The same or no letters indicate non-significant differences (p>0.05).

**Table 3:** Comparison of sequenced transcriptome sequences with reference sequences

Sample	Raw reads	Clean reads	Error rate (%)	Q20 (%)	Q30 (%)	GC content (%)	Total map (%)
M8_H_1	44434682	42528134	0.03	96.54	90.61	49.99	96.08
M8_H_2	42283360	40151204	0.03	97.8	93.7	48.82	96.37
M8_H_3	44388700	42448198	0.03	97.86	93.96	52.15	96.63
M8_EH_1	46805968	44017458	0.03	97.86	93.47	50.55	96.17
M8_EH_2	45300712	42033122	0.03	97.85	93.81	49.13	96.5
M8_EH_3	46078322	43830296	0.03	97.88	93.97	51.75	96.59
M8_SHE_1	45587106	43441966	0.03	97.83	93.77	48.17	96.45
M8_SHE_2	44886924	42725478	0.03	97.62	93.49	53.86	95.78
M8_SHE_3	45410674	43989632	0.03	97.7	93.57	52.12	96.68
Average	45019605	42796165	0.03	97.66	93.37	50.73	96.36

GC, guanine-cytosine; M8_H, 8-month-old Hu sheep; M8_EH, 8-month-old East Friesian×Hu sheep; M8_SHE, 8-month-old Suffolk sheep×EH.
